# Enhanced Skin Permeation and Controlled Release of β-Sitosterol Using Cubosomes Encrusted with Dissolving Microneedles for the Management of Alopecia

**DOI:** 10.3390/ph16040563

**Published:** 2023-04-08

**Authors:** Kousalya Prabahar, Ubaidulla Uthumansha, Nehal Elsherbiny, Mona Qushawy

**Affiliations:** 1 Department of Pharmacy Practice, Faculty of Pharmacy, University of Tabuk, Tabuk 71491, Saudi Arabia; 2 Department of Pharmaceutics, Crescent School of Pharmacy, B.S. Abdur Rahman Crescent Institute of Science and Technology, Chennai 600048, India; 3 Department of Pharmaceutical Chemistry, Faculty of Pharmacy, University of Tabuk, Tabuk 71491, Saudi Arabia; 4 Department of Biochemistry, Faculty of Pharmacy, Mansoura University, Mansoura 35516, Egypt; 5 Department of Pharmaceutics, Faculty of Pharmacy, University of Tabuk, Tabuk 71491, Saudi Arabia; 6 Department of Pharmaceutics, Faculty of Pharmacy, Sinai University, Alarish 45511, North Sinai, Egypt

**Keywords:** cubosomes, microneedles, beta-sitosterol, alopecia, dermal drug delivery, testosterone induced alopecia animal model, ex vivo skin permeation

## Abstract

The use of synthetic medication for treating alopecia is restricted because of systemic exposure and related negative effects. Beta-sitosterol (β-ST), a natural chemical, has lately been studied for its potential to promote hair development. The cubosomes with dissolving microneedles (CUBs-MN_D_) created in this study may be a useful starting point for the creation of a sophisticated dermal delivery system for β-ST. Cubosomes (CUBs) were prepared by the emulsification method, using glyceryl monooleate (GMO) as a lipid polymer. CUBs were loaded with dissolving microneedles (MN_D_) fabricated with HA and a PVP-K90 matrix. An ex vivo skin permeation study and an in vivo hair growth efficacy test of β-ST were performed with both CUB and CUB-MN_D_. The average particle size of the CUBs was determined to be 173.67 ± 0.52 nm, with a low polydispersity index (0.3) and a high zeta potential value that prevents the aggregate formation of dispersed particles. When compared to CUBs alone, CUBs-MN_D_ displayed higher permeating levels of β-ST at all-time points. In the animals from the CUB-MN_D_ group, significant hair development was observed. According to the results of the current investigation, CUBs that integrate dissolving microneedles of β-ST are superior in terms of transdermal skin penetration and activity for the treatment of alopecia.

## 1. Introduction

Topically administered drugs are typically chosen over those taken orally or injected intralesionally for the treatment of alopecia due to the reduced potential for systemic exposure and associated side effects [1,2]. Furthermore, 5-Alpha-reductase is the enzyme responsible for turning testosterone into dihydrotestosterone (DHT), and it is mostly located in hair follicles on the scalp [3,4]. Numerous scientific investigations have established that balding scalp follicles contain a significantly higher concentration of DHT [5]. The stratum corneum is a lipid bilayer composed of keratinocytes that are 10–15 μm thick, making it impenetrable to most available traditional topical dose forms [6]. Adapting different medication compounds for topical application was hampered by this obstacle. Therefore, a unique topical medication delivery platform is needed for the treatment of alopecia. Microneedles (MNs) have gained significant attention in recent years for drug administration at the surface of the skin because of their potential to circumvent this obstacle and bring medication to its intended location [7].

MNs can be classified into several different types, including those that are coated, porous, hydrogel, hollow, and dissolving [8]. Dissolving microneedles (MN_D_s) are a type of microneedle device that can release the encapsulated medicine into the skin more quickly than other methods [9]. This could prevent potentially dangerous items from being implanted under the skin because the polymers of the needles break down entirely after insertion [10]. Indeed, the use of polymers such as polyvinyl pyrrolidone (PVP), hyaluronic acid, poly (methyl vinyl ether-co-maleic anhydride), carboxy methyl cellulose, and polysaccharides to create MN_D_s has been extensively documented [11]. 

Lipophilic beta-sitosterol (β-ST) is a naturally occurring phytosterol molecule that has been used to cure alopecia [12]. Possible β-ST mechanisms include early hair follicle transition from the telogen to the anagen phase and the inhibition of 5α-reductase [13,14]. As β-ST has low absorption and requires a high dose (i.e., up to 3 g/day) when administered orally, the topical route of administration is preferred. The hair growth rate of alopecia-induced rats was dramatically increased after treatment with phytovesicles laden with β-ST, an approach which was developed by Upadhyay et al. (2012) [15]. Cubosomes have been advocated as a carrier for topical drug delivery due to their various benefits over liposomes. These benefits include a high entrapment efficiency, controlled release, bioadhesiveness, and biosimilarity to the epithelial cells of the skin [16]. Controlled release nanomedicine delivery, however, is a translational therapeutic method for managing alopecia due to the improved retention of the medications inside skin layers [17]. 

According to Hosny et al. [18], who prepared nano cubosomes loaded with finasteride for the treatment of alopecia, the cubosomes improved skin penetration and regulated the release of finastride [18]. Curcumin-loaded cubosomes were created by Victorelli et al. for topical distribution, and they were identified as a very promising platform for the addition of lipophilic medications [19]. The goal of the current study was to create β-ST-loaded cubosomes. The use of MN-mediated transdermal administration has been investigated for increasing the skin penetration of β-ST-loaded cubosomes. MN-aided nanocarriers are a promising method for the creation of cutting-edge drug delivery systems that aim to increase the effectiveness of alopecia medication treatments.

To the best of our knowledge, there are currently no papers available that discuss the use of dissolving, MNs-mediated, β-ST-loaded cubosomes as a therapy for alopecia. This dual delivery method, which combines the vesicular system and MNs, has the potential to increase the drug’s effectiveness through the use of cubosomes, facilitate the drug’s passage through the skin barrier via MN pores, and provide further control over its release. In this study, MN_D_-loaded cubosomes were prepared and analyzed using XRD and FTIR. Particle size, zeta potential, scanning electron microscopy (SEM), and drug content were also examined. The ability to penetrate the skin, the in vitro drug release, the ex vivo skin permeation investigation, and the stability of the compound were all evaluated.

## 2. Results and Discussion

Five formulations of β-ST-loaded cubosomes were prepared by a homogenization and sonication method and evaluated for EE%, particle size, and in vitro release. 

### 2.1. Inspection of the Prepared β-ST-CUBs

The β-ST-loaded cubosomes were prepared using GMO and stabilizer Pluronic F-127, and transparent gels were obtained except when the ratios of GMO to β-ST were 60:10. Table 1 shows the formulation compositions and the following CUBs, FC2, FC3, and FC4, were selected for further characterization. The results corroborate well with earlier reports. The transparent gels are believed to be cubic phases since the cubic phase is known to be isotropic and optically transparent [20]. FC5 formulation showed an opaque white gel layer, which may be attributed to the fact that β-ST is a lipophilic and oil-soluble drug and the cubic phase would accommodate more drug molecules in its hydrocarbon chain matrix, leading to the formation of another phase which an opaque white gel layer [21]. 

### 2.2. Particle Size, Polydispersity Index, and Zeta Potential of β-ST-CUBs

As shown in Table 2, the average particle sizes of the β-ST-loaded cubosomes FC2, FC3, and FC4 were 265.03 ± 0.71 nm, 224.41 ± 0.97 nm, and 173.67 ± 0.52 nm, respectively, with a small polydispersity index (<0.3), which indicates a narrow size distribution. This result revealed that the solubilization of β-ST in lipid matrix occurred well and formed a homogeneous micelle size [22]. 

The hydrophilic copolymer (Pluronic F127) led to an increase in its ability to break down large aggregates, forming smaller particles with more uniform size distribution. More heterogeneous lipid-based vesicles were reported when the concentration of Pluronic F127 was increased. In high amounts, Pluronic produced diverse morphologies in the cubosomal dispersion that was translated into a high PDI value.. 

These different morphological components can arise from the formation of both micelles and lipid/Pluronic F127 mixed aggregates. Homogenous lamellar structures could only be formed at low Pluronic concentrations, while higher concentrations resulted in self-aggregation and the initiation of multiple forms, making the dispersion more heterogonous [23].

As represented in Table 2, all ZP values of the formulated nano-structured cubosomal systems were negative. The range of ZP of the β-ST-loaded CUBs was found to be from −28.67 ± 0.34 to −35.88 ± 1.32 mV, which is in accordance with the theoretically accepted values of ZP [24,25].

The surface negative charge may be due to the hydroxyl groups of the stabilizer (Pluronic F-127), and this negative charge might also be attributed to the ionized carboxylic end group in the free fatty acids (GMO), usually free oleic acid, present within the GMO and adsorbed on the cubosomes due to the lipophilic nature of these acids [26]. It is documented that ZP values of approximately ± 30 mv are required to ensure the stability of colloidal dispersions [27]. This value provides an electrostatically thick diffusion layer that acts as a boundary, preventing dispersed particles from being aggregated [25]. 

### 2.3. The Entrapment Efficiency Determination of β-ST-CUBs

The encapsulation efficiency % of the CUB formulations FC2, FC3, and FC4 were calculated as 92.18 ± 0.97%, 95.04 ± 0.75%, and 98.13 ± 0.61%, respectively (*n* = 3). These results revealed that the CUBs accommodated the highest amount of drug due to the large surface area of the cubic phase and the lipophilic property of β-ST, which can distribute within lipid layers, resulting in a higher encapsulation efficiency of the CUBs.

### 2.4. The In Vitro Release Study of β-ST-CUBs

**Figure 1:**
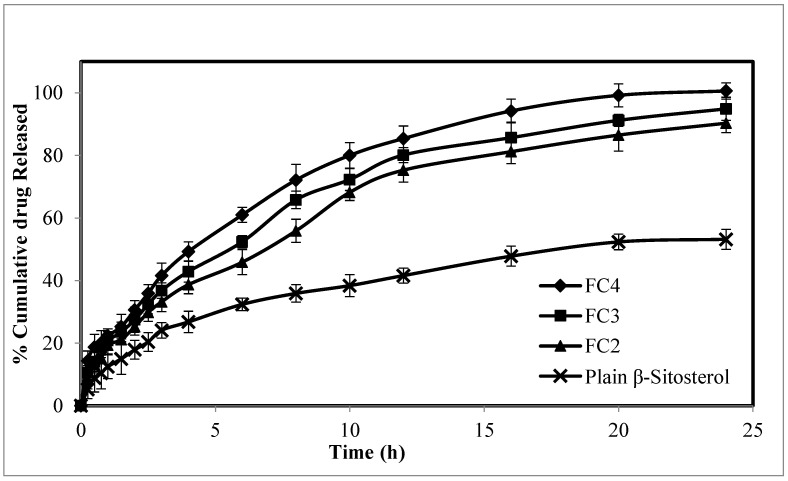
In vitro release study of β-ST from the prepared β-ST-CUBs.

As shown in Figure 1, all β-ST-CUBs showed a biphasic release pattern, with an initial burst followed by sustained β-ST release for 24 h. This result revealed that the initial burst effect may be attributed to the drug adsorbed on the cubosome surface, while the sustained release performance may be attributed to the drug entrapped inside the cubosomes [28,29]. The β-ST release profiles from the cubosome particles showed that the drug released faster from formulation FC4 (99.8%) than from formulation FC2 and FC3, approximately 90.3% and 94.89% at 24 h, respectively. 

Increasing the GMO concentration resulted in decreasing the drug release rate from the cubosomes. An increase in the GMO concentration increases the matrix viscosity, and this will, in turn, retard drug diffusion from the lipid bilayer to the aqueous release medium and eventually slow the drug release rate [30]. In turn, this supports the slowest release results obtained by the FC2 and FC3 cubosomal formulations with the highest % lipids due to the hindered diffusion of the release medium to the center of cubosome and hindered drug diffusion outside the cubosome.

The slower release of the β-ST suspension in comparison with the β-ST cubosomes may be attributed to the effect of nanoencapsulation on increasing drug solubility. Hence, a higher amount of drugs were released from the nanoformulation in comparison with the drug suspension. This result corroborates well with the earlier findings of Mariam Zewail et al., 2022 [30].

At higher concentrations of GMO, the topical delivery of β-ST was still increased, but its transdermal delivery was substantially reduced. Due to their lipophilic nature, glycerol monooleate and β-ST are likely to have a high affinity to each other. The interaction between the glycerol monooleate (at high concentration) and β-ST may result in drug retention in the skin where glycerol monooleate is better partitioned. Incorporated drug molecules can be released in a sustained manner due to the huge interfacial area, which offers complex diffusion pathways. This may be due to lipophilic drugs being positioned inside the lipid bilayer [31]. Additionally, their lipidic contents are biocompatible and bioadhesive, which promotes them as a topical delivery carrier for β-ST. 

#### In Vitro Release Kinetic Analysis

**Figure 2:**
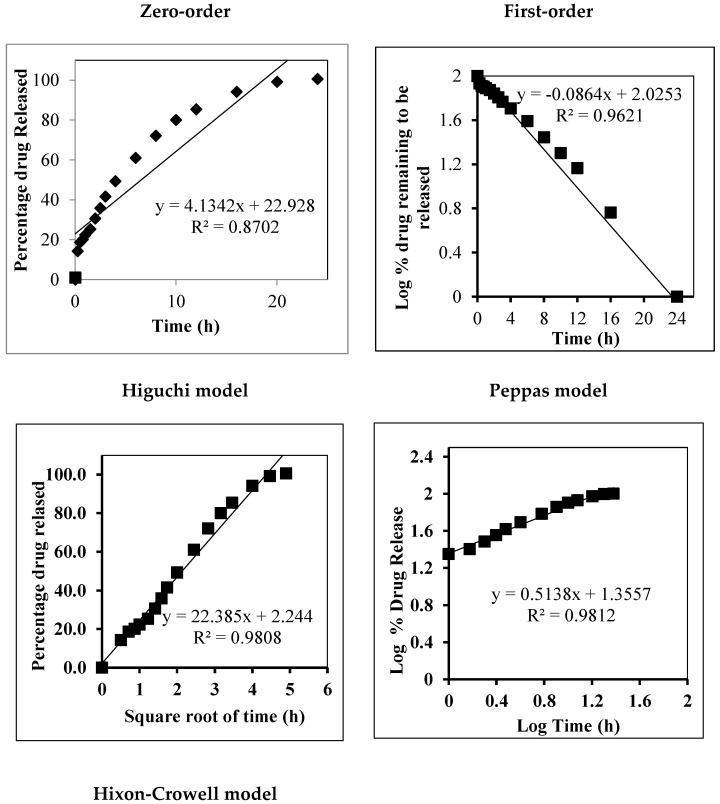

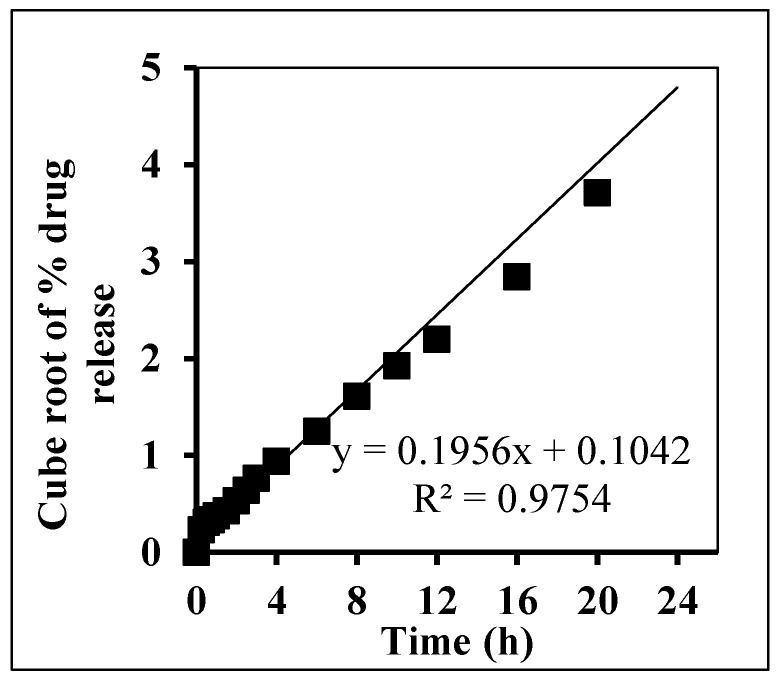
The release kinetics of optimized, β-ST-loaded cubosomal formulation (FC4).

The release kinetics of the optimized, β-ST-loaded cubosomal formulation (FC4) were fitted to the zero, first, Higuchi, Peppas, and Hixon–Crowell models, and their correlation coefficients (R^2^) are shown in Figure 2. The results show that Higuchi’s model demonstrated an R^2^ value of 0.9808, suggesting that the β-ST release from cubosomes occurred by a diffusion mechanism.

The best fit was obtained for the Korsmeyer–Peppas model, which was first proposed to describe different drug release mechanisms from polymeric films and is described by the following equation:

Q = k⋅t*^n^* (1)

where Q represents the proportion of β-ST released at time (t), k represents the release rate constant, and *n* represents the release exponent that typifies the drug release mechanism: Fickian diffusion is indicated by *n* = 0.43 and zero-order release is indicated by *n* = 1, while 0.43 < *n* <  1 values are related to anomalous transport. The obtained value of *n* = 0.5138 suggests that drug release follows a non-Fickian or anomalous transport in which the rate of β-ST release from the cubosomes is governed by both drug diffusion and polymer relaxation (erosion). This result is in good agreement with an earlier study of etodolac-loaded cubosomes [32].

The slow release and high drug encapsulation efficiency are desirable as they can reduce the volume of dosage form required to achieve the desired therapeutic effect, which indicates that the β-ST-loaded cubosomes are most beneficial for the treatment of alopecia.

### 2.5. Transmission Electron Microscopy of Optimized β-ST-CUB

**Figure 3:**
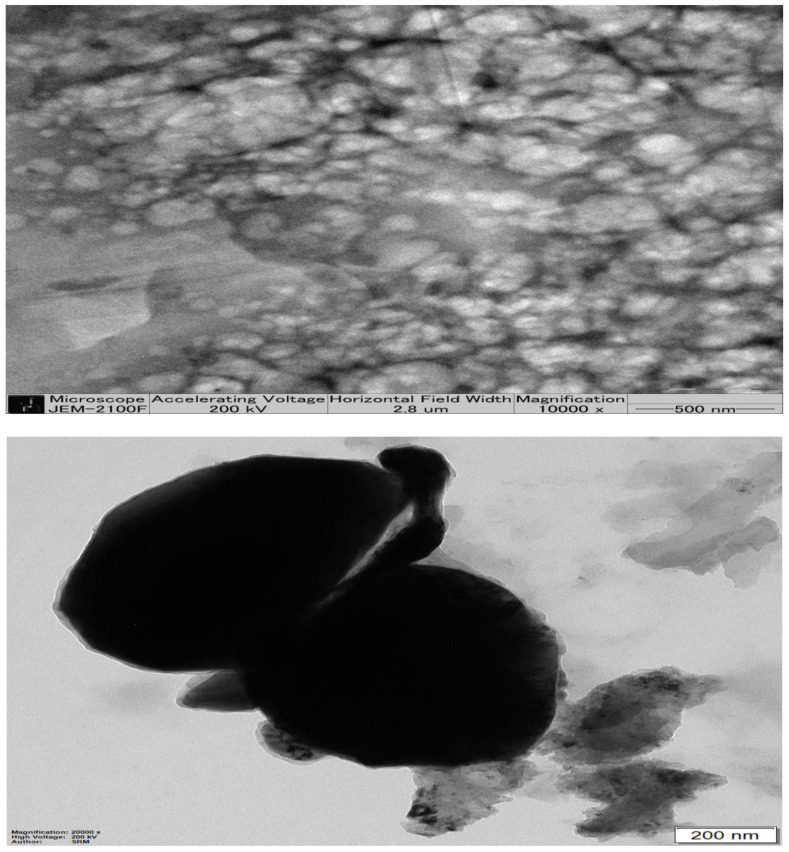
TEM images of optimized β-ST-loaded Cubosomes.

As shown by Figure 3, a TEM image of β-ST-loaded cubosomes shows a smooth surface and hexagonal shape. The vesicles appear as non-aggregated particles with a homogenous and narrow size distribution. However, the diameters of TEM-photographed particles are smaller relative to the measured particle size obtained by the dynamic light-scattering particle size analyzer. 

### 2.6. X-ray Diffraction of β-ST-CUBs

**Figure 4:**
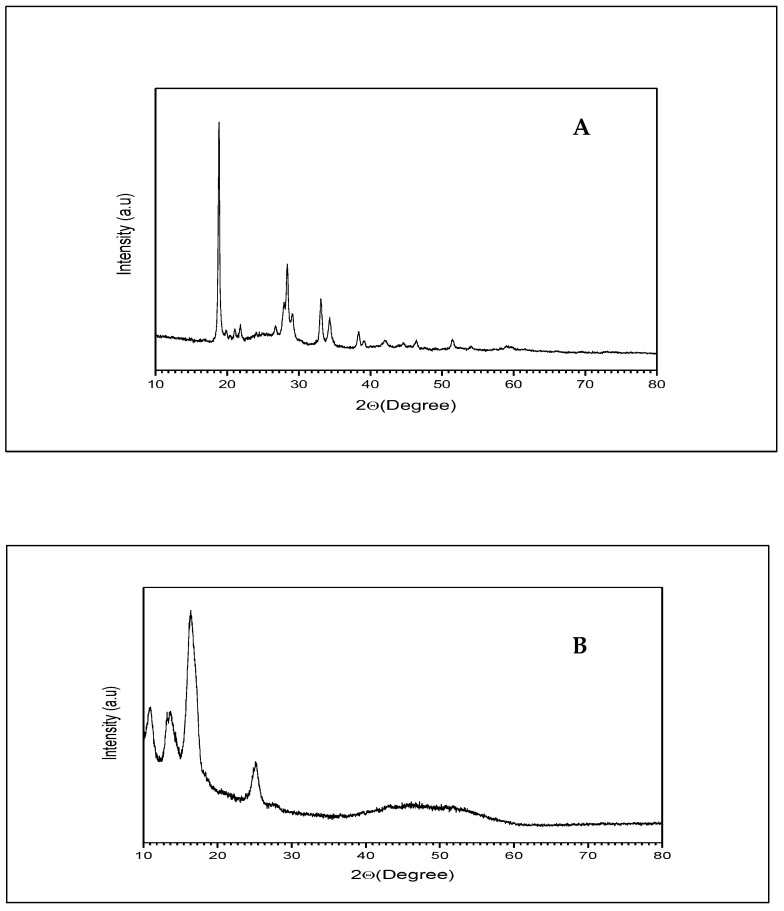
X-ray diffraction of (**A**) β-ST and (**B**) β-ST-loaded cubosomes.

The X-ray diffraction of β-ST and β-ST loaded cubosomes is shown in Figure 4. β-ST diffraction showed specific sharp peaks with a high intensity, indicating the crystalline nature of β-ST. On the other hand, the β-ST-loaded cubosomes did not show peaks of high intensity, indicating the presence of β-ST in the amorphous state in the bicontinuous structure of the cubosomes. Similar results were obtained by Nasr et al., who found that the X-ray diffraction of 5-Fluorouracil cubosomes revealed the presence of the drug in the non-crystalline form [33]. 

### 2.7. Characterization of β-ST-Loaded CUBs-MN_D_

**Figure 5:**
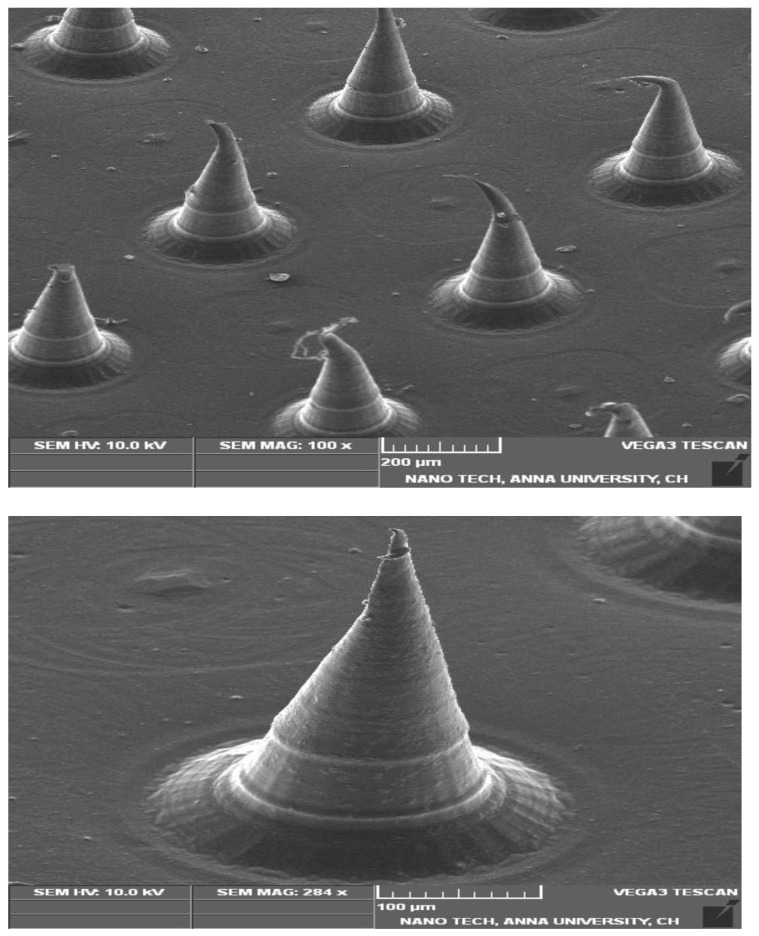
SEM images of the prepared β-ST-loaded CUBs-MN_D_.

The prepared MN_D_s, loaded with β-ST-CUBs, were evaluated regarding the diameter of the tip, the diameter of the base, and the length of CUB-MN_D_ arrays, and the aspect ratio, using optical microscope. As represented in Table 3, it was found that the length of the CUBs-MN_D_ was 621.34 ± 15.70 µm, the tip diameter was 27.25 ± 4.17 µm, the base diameter was 328.82 ± 18.39 µm, and the aspect ratio was 0.86 ± 0.08. Regarding the drug content, it was found that the drug content of the CUBs-MN_D_ was 93.79 ± 2.55%. Figure 5, shows the SEM image of the prepared CUBs-MN_D_. 

### 2.8. In Vitro Skin Permeation Study of β-ST from CUBs and CUBs-MN_D_

**Figure 6:**
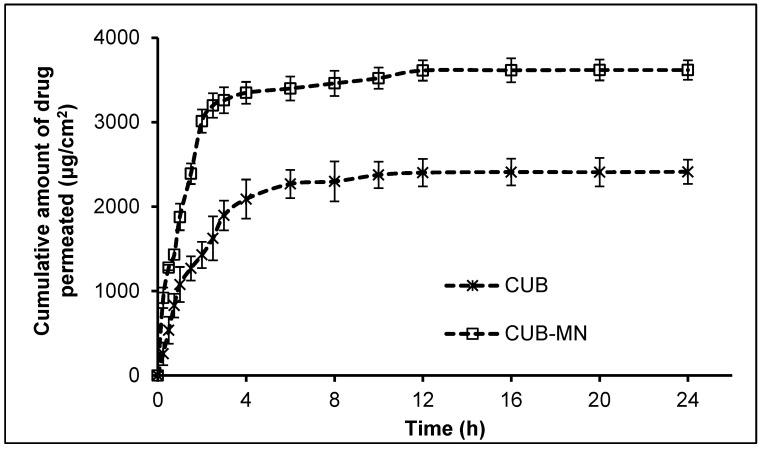
In vitro release of β-ST from CUBs and CUBs-MN_D_.

The permeation of β-ST from the prepared CUBs-MN_D_ was studied over 24 h using an ex-vivo skin membrane, see Figure 6. The steady state flux (Jss) of the CUBs and CUBs-MN_D_ was determined from the slope of the linear portion of the cumulative drug release plots, and the result was found to be 82.818 and 101.17 μg/cm^2^/h respectively. The result revealed that the CUBs-MN_D_ rapidly dissolved in the skin’s extracellular fluid after application, and the drug was released from the liberated CUBs in a controlled manner and finally reached the target site. During this stage, the CUBs diffused and/or partitioned into the specific dermal layer while releasing the drug sustained their rate. The dissolving microneedles utilizing HA and PVP-K90 showed a faster release of β-ST into the skin due to the microneedle matrix dissolving rapidly and avoided the diffusion step from outside the skin by MN_D_ [34].

### 2.9. The Stability Study of CUBs-MN_D_

During the stability studies, the formulations were monitored at 25 ± 2 °C (60 ± 5% RH), 40 ± 2 °C (75 ± 5% RH), and 5 ± 2 °C, and their physical characteristics, drug content, and ex vivo drug permeation patterns were assessed after 6 months. As represented in Table 4, there was no change in the physical appearance of the microneedles, the % drug content, and the drug permeation profiles, which showed the stability of the CUBs-MN_D_ under all storage conditions. From the results, it can be concluded that the microneedle patches remained stable under all storage conditions during the storage period of 6 months.

### 2.10. The IR Spectroscopy

**Figure 7:**
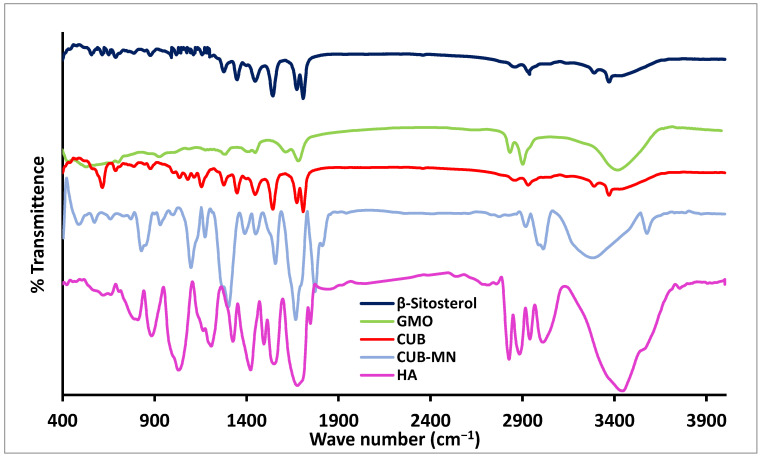
IR spectra of β-ST, GMO, HA, CUBs, and CUBs-MN_D_.

IR spectroscopy is one way to determine the compatibility of a drug with other substances. As shown in Figure 7, the characteristic FT-IR Absorption of β-ST was shown at 3328 cm^−1^ (stretching vibration of OH), 2990 cm^−1^ (stretching vibration of CH alkane (symmetric and asymmetric)), 1640 cm^−1^ (stretching vibration of C=C), 1580 cm^−1^ (bending vibration of OH), 1460 cm^−1^ (bending vibration of isopropyl), and 1312 cm^−1^ (bending vibration of C-O of 2° alcohol). These results were in agreement with Azeez et al., who studied the IR spectrum of β-ST [35]. As shown in Figure 7, the characteristic peaks of GMO appeared at 3392 cm^−1^, corresponding to OH bond, 2929 cm^−1^ corresponding to C-H stretching, and 1741 cm^−1^ due to stretching C=O. Additionally, HA showed its characteristic peaks at 3348 cm^−1^ due to the presence of OH stretching and N-H stretching vibrations in the N-acetyl side chain. The IR spectrum of HA also showed a peak at 2927 cm^−1^ due to the symmetric methyl C-H stretch group of glucuronic acid, and two peaks at 1637 and 1421 cm^−1^ due to C=O carboxyl and primary aromatic amine CN stretching, respectively. Similar results were obtained by Mohamed et al., who studied the IR spectrum of HA [36]. All characteristic peaks of the β-ST bands appeared in the CUBs and CUBs-MN_D_, indicating that there was no chemical interaction between the drug and excipients. 

### 2.11. Hair Growth Effect of β-ST-Loaded CUBs-MN_D_

**Figure 8:**
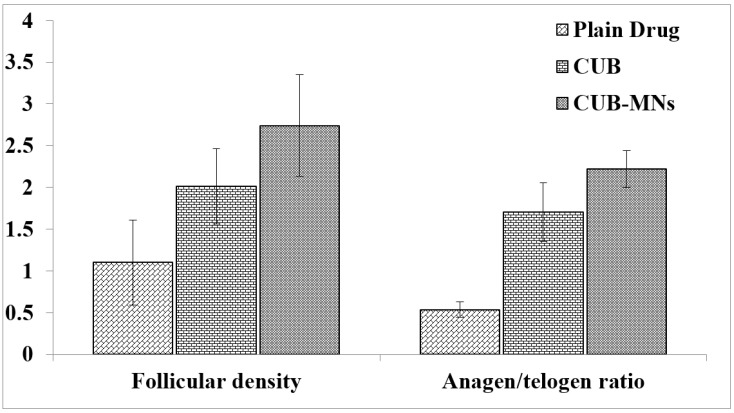
Hair growth pattern in CUB-MN_D_-treated groups was comparable to that for the β-ST-loaded CUB and plain drug groups.

**Figure 9:**
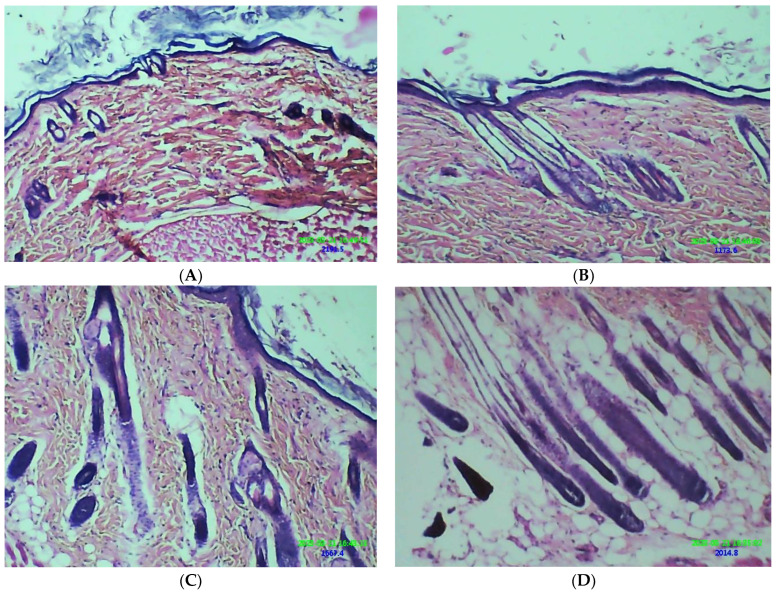
Comparison of hair regrowth pattern in rat skin histogram of following groups; (**A**) testosterone solution only; (**B**) testosterone with β-ST gel; (**C**) testosterone + CUBs; and (**D**) testosterone + CUBs-MN_D_.

The hair-growth efficacy of β-ST in different treatment approaches is shown in Figure 8 and Figure 9. The CUB-MN_D_ group showed significant hair growth (*p* < 0.05) among all other groups. This may be because the CUBs’ embedded, dissolving microneedles may serve as a reservoir, slowly releasing the β-ST at the insertion site and enhancing drug concentration [22]. 

Moreover, the HA polymer is biocompatible, bioerodible, and rapidly dissolved in the skin’s extracellular fluid, facilitating the microneedles’ system for improving the permeation of the β-ST [37]. The slow release and high drug encapsulation efficiency is desirable as they can reduce the volume of the dosage form required to achieve the desired therapeutic effect, indicating that the β-ST-loaded cubosomes that incorporate microneedles are the most beneficial for the treatment of alopecia.

## 3. Materials and Methods

### 3.1. Materials 

β-ST, hyaluronic acid, and Poloxamer 407 (Pluronic F-127) were purchased from Sigma Aldrich, USA. Glyceryl monooleate (GMO) received as a gift from Masterowin Pharmaceuticals, India. Polyvinylpyrrolidone (PVP) –K 30 was purchased from Spectrum Chemical, USA. Methanol and acetonitrile were bought from Merck, India. Distilled water was obtained from an inner source. All other chemicals and reagents used in the study were of analytical grade.

### 3.2. Design and Preparation of Beta-Sitosterol Cubosomes (β-ST-CUBs)

β-ST-loaded cubosomes were produced utilizing an earlier technique that is primarily designed for lipid-soluble drugs [21]. Glyceryl monooleate (GMO) and Pluronic F-127 were first completely melted at 60 °C in a hot water bath. β-ST was then added to dissolve/blend under constant stirring [38]. Drop by drop, distilled water was then added, and the mixture was vortexed to obtain homogeneity. The cubic phase gel was obtained after 48 h of equilibration at room temperature [39]. The cubic gel was broken up by mechanical stirring after just 20 mL of water was added. The gels were subsequently micronized in a probe-type sonicator (Enertech Electronics, Chennai, India) for 15 min at room temperature using a cycle of 30 s pulse on, 30 s pulse off [40]. A high-shear homogenizer (Remi, India) was then used to homogenize the resulting milky, coarse dispersion at 2000 revolutions per minute to yield an opalescent dispersion of the cubic nanoparticles. For subsequent research, the liquid crystal nanoparticles’ final dispersion was kept at room temperature.

### 3.3. Characterization of CUBs

#### 3.3.1. Determination of the Particle Size, Polydispersity Index (PDI), and Zeta Potential (ZP) of β-ST-CUBs

The particle size, polydispersity index (PDI), and Zeta potential (ZP) of the prepared β-ST-CUBs formulations were determined by dynamic light scattering technique using a Malvern^®^ Zetasizer Nano ZS90 (Malvern^®^ Instruments Limited, Worcestershire, UK). Each formulation was subjected to measurement after dilution by double-distilled water (1:200). The measurements were performed in triplicate using a 90° scattering angle [41].

#### 3.3.2. Determination of the Entrapment Efficiency (EE%) of β-ST-CUBs

An aliquot (2 mL) of CUBs was centrifuged for 45 min at 7000 rpm to determine the drug encapsulation efficiency (EE), using an indirect method [42,43]. To quantify the free drug in the clear supernatant, reverse-phase high-performance liquid chromatography (RP HPLC) technology was used using a Waters 2690 Alliance HPLC system (Waters, Milford, MA, USA). The HPLC separation was carried out using a 20 µL injection volume and a mobile phase of methanol and acetonitrile (at a ratio of 30:70 *v*/*v*). A flow rate of 1 mL/min was used to elute the samples while the eluent was monitored at a wavelength of 210 nm. The effectiveness of β-ST encapsulation was calculated using the following equation.

$$EE\%\;=\frac{Total\;amount\;of\;beta\;sitosterol-Unentrapped\;beta\;sitosterol\;}{Total\;amount\;of\;beta\;sitosterol}\;\times 100$$

#### 3.3.3. In Vitro Release Study of β-ST from the Prepared β-ST-CUBs

The dialysis bag approach was utilized in order to carry out an in vitro drug release investigation on the β-ST derived from the prepared CUB formulations [22]. After being thoroughly cleansed, dialysis membranes with a cut-off of 14,000 Da were left to spend the night soaking in the dissolving medium. After the bags were filled with a β-ST-loaded CUB suspension, they were subsequently submerged in a 50 mL phosphate buffer (pH 7.4) containing 2.5% Tween 80 at 37± 1 °C while being stirred at 100 rpm [44]. The data from the samples, which were obtained at regular intervals of time, were analyzed using the HPLC method to determine the amount of β-ST that was released.

The in vitro drug release data were fitted with different mathematical models, such as zero, first, Higuchi, and Korsmeyer–Peppas, to analyze the release kinetic pattern [45]. 

#### 3.3.4. Transmission Electron Microscopy (TEM)

A transmission electron microscope was used to determine the form and surface morphology of the optimized CUBs. Specimens were made by drop-casting the cubosomes on a copper grid with 10% phosphotungstic acid [46,47]. After being deposited onto a carbon-coated grid with a mesh size of 200, the stained cubosome suspensions were allowed to air-dry at room temperature. A transmission electron microscope (JTEM model 1010, JEOL, Tokyo, Japan) was used to capture the images [48]. 

#### 3.3.5. X-ray Diffraction Study

The crystalline behavior of the β-ST-CUBs was evaluated with X-ray diffractometry in comparison to pure β-ST. Diffraction patterns were obtained on RIGAKU MINI FLEX II- Japan [49]. The X-ray generator was operated at a 40 kV tube voltage and a 35 mA tube current [33].

### 3.4. Preparation of β-ST-CUBs Loaded with Dissolving MN_D_

β-ST-CUBs loaded with dissolving MN_D_ were manufactured utilizing a modified version of the original process. Two-step casting was used to create dissolving microneedles utilizing HA and PVP-K90 in a micro-molding process [34]. In brief, the CUBs’ tip solution was combined with a polymeric solution of HA in distilled water. Separately, the backing solution was produced with an aqueous polymeric solution of PVP-K90. The β-ST-CUBs polymeric solution was placed into the polydimethylsiloxane (PDMS) mold and centrifuged at 4000 rpm for five minutes. After draining the excess solution, the PDMS mold was left overnight to dry in a silica gel desiccator. The PDMS mold was then filled with PVP solution and centrifuged at 4000 rpm for 10 min [50]. Later, the mold was transported into the chamber under positive pressure, and the pressure was then progressively removed. The silicone molds were then cured at 37 °C for 24 h. MN_D_s were extracted from the molds and examined visually for effective needle creation. The β-ST-loaded CUBs that had been absorbed into the dissolving MNs were then carefully removed from the molds and stored in a desiccator for subsequent analysis.

#### 3.4.1. Evaluation of CUB-MN_D_

##### Morphological Studies

Using a microscope, the diameter of the tip, the diameter of the base, and the length of the CUB-MN_D_ arrays were determined. The aspect ratio was determined by the length-to-base diameter ratio. All measurements were performed in triplicate, and the mean ± SD was obtained [51].

##### Drug Content

An array of CUBs-MN_D_ was allowed to be dissolved in distilled water containing 2.5% Tween 80 by stirring at 300 rpm using a magnetic stirrer for 1 h. The resultant solution was mixed with methanol for dilution and subjected to sonication for 5 min to allow the complete dissolution of β-ST from the fabricated CUBs-MN_D_ [52]. The drug content in the prepared MN_D_s was determined by analysis using HPLC.

##### Scanning Electron Microscopy of CUBs-MN_D_ (SEM)

The microneedle patch morphology was examined for needle shape, size, and other physical characteristics using SEM (JEOL-JSM5600, Tokyo, Japan) [53]. The samples were coated with gold solution, operating at 10 kV for 15 s under a low vacuum to obtain a better contrast [54].

##### In Vitro Skin Permeation Study

A Franz diffusion cell apparatus (Electrolab India pvt Ltd., Navi Mumbai, Maharashtra, India) was used in in vitro permeation tests for the CUB-MN_D_ formulations [55]. Male Wistar rats (weighing 200–250 g) with full-thickness abdomen skin were used. The experimental protocol was approved by the institutional animal ethical committee (IAEC Approval No. 04/321/PO/Re/S/01/CPCSEA). An electric clipper was used to delicately shave the area around the abdomen. With distilled water, the skin was thoroughly cleaned of any clinging tissues or blood vessels. Before starting the experiment, the skin was immersed in phosphate buffer pH 7.4 for 1 h. The isolated rat skin piece was positioned with the epidermis facing above and the dermis layer facing downward between the donor and receptor compartment of Franz diffusion cells [56]. The rat skin was exposed to transdermal microneedle patch formulations (CUBs-MN_D_) [57]. To maintain the sink condition, the diffusion medium was maintained at 37 ± 0.5 °C, stirred at 100 rpm, and provided with Tween 80 (2.5%). The sample (1 mL) was withdrawn from the receptor compartment and replaced with an equal volume of fresh medium at predetermined intervals. The sample’s content of β-ST was determined by HPLC, and the cumulative amount of drug that permeated throughout time was plotted versus time. The slope of the linear part of the cumulative drug release plots was used to calculate the steady state flux (Jss) of the CUBs and CUBs-MN_D_ [58].

##### Stability Testing of CUB-MN_D_

The β-ST loaded cubosomes incorporated dissolving microneedles (CUBs-MN_D_) were maintained under three different conditions: 25 ± 2 °C (60 ± 5% RH); 40 ± 2 °C (75 ± 5% RH); and 5 ± 2 °C for 6 months to determine the stability of the microneedle mediated transdermal system. 

##### Fourier-Transform Infrared (FTIR) Study

The compatibility between drug and other ingredients can be studied using Fourier-transform infrared (FTIR). Each sample (β-ST, GMO, HA, CUBs, and CUBs-MN_D_) was compressed into a disc in presence of potassium bromide. The compressed discs were scanned from 4000 to 400 cm^−1^ using a Shimadzu 435 U-O4 IR spectrometer (Tokyo, Japan) [59]. 

### 3.5. Animals

Male Wistar rats weighing 230–250 g were obtained from the C.L. Baid Metha College of Pharmacy’s Central Animal House in Chennai, India. The animals were housed in a typical laboratory setting with a temperature of 25 °C and a relative humidity adjusted at 55%. The animals were housed in polypropylene cages that were separated into five groups, each with six animals. They received unlimited water and ate a standard laboratory diet (Lipton feed, Mumbai, India). The Institutional Animal Ethical Committee authorized the experimental protocol (IAEC Approval no: 04/321/PO/Re/S/01/CPCSEA).

#### Animal Groups and Treatments

The method reported by Upadhyay et al. was followed with minor changes [15]. Five groups of animals, each with six animals, were prepared. Depilatory cream was used to remove the dorsal hair of rats (2 by 2.5 cm). The following animal groups were given different treatments: (A) Intact control (did not receive testosterone); (B) testosterone solution only; (C) testosterone with β-ST gel; (D) testosterone + CUBs; and (E) testosterone + CUBs-MN_D_: the patch was firmly pressed for 30 s to penetrate through the epidermis and then pressed softly for an extra 2 min. The patch base was peeled at 10 min post insertion into the skin, allowing the MNs to settle in the skin for further sustained drug release. Except for group A, all other groups were administered testosterone (0.5 mg/Kg/Daily) subcutaneously (SC), and Groups C, D, and E received β-ST (1 mg/Kg/Daily) topically for 21 days. The hair growth was observed by qualitative evaluation. After 21 days, the difference in hair growth in each group was examined visually and photographed. A quantitative analysis of hair growth was performed. After shaving the long hair, the skin of the animals in the dorsal area was dissected and fixed in 10% formalin. Vertical sections of the skin were prepared after fixation and stained with hematoxylin and eosin (H&E). To evaluate hair growth, the sections were analyzed for various parameters. The follicular density (number of follicles/mm) was reported by recording the number of hair follicles in a 2 mm area. The anagen/telogen ratio was determined by calculating the number of follicles in the anagen phase (active growth phase) and telogen phase (resting phase).

### 3.6. Statistical Analysis

Data were reported as the mean ± SD. Data were analyzed by one-way analysis of variance (ANOVA) followed by Tukey’s post hoc test. *p* < 0.05 was considered significant.

## 4. Conclusions

In this study, dissolving, MNs-mediated β-ST-loaded cubosomes were prepared successfully. The ex vivo skin permeation study confirmed that the CUBs-MN_D_ showed higher permeating amounts of β-ST at all-time points when compared with the CUBs alone. Significant hair growth was observed in testosterone-induced alopecia animals after the treatment with CUBs-MN_D_. Therefore, CUBs-MN_D_ may contribute to improved administration regimens by increasing the concentration of the drug in the skin and decreasing the dosing frequency, leading to enhanced hair-growth efficacy of β-ST. However, further human clinical trials with alopecia populations are still necessary to highlight the practical application for MN_D_ drug delivery.

## Figures and Tables

**Table 1:** The composition of β-ST-CUBs.

Formulation Code	GMO (%)	β-ST (%)	Pluronic F-127 (%)	Water (mL)	Appearance
FC 1	100	-	10	10	Transparent
FC 2	90	10	05	10	Transparent
FC 3	80	10	10	10	Transparent
FC 4	70	10	15	10	Transparent
FC 5	60	10	15	10	Opaque white

**Table 2:** The measured particle size, PDI, and ZP of the prepared β-ST-loaded CUB.

Formulation Code	Particle Size (nm)	PDI	ZP (mV)
FC 2	265.03 ± 0.71	0.187 ± 0.01	−28.67 ± 0.34
FC 3	224.41 ± 0.97	0.267 ± 0.02	−31.27 ± 1.48
FC 4	173.67 ± 0.52	0.285 ± 0.02	−35.88 ± 1.32

**Table 3:** Physical characteristics of the CUBs- MN_D_.

Physical Characteristics (*n* = 20)	
Length (μm)	621.34 ± 15.70 μm
Base diameter (μm)	328.82 ± 18.39 μm
Tip diameter (μm)	27.25 ± 4.17 μm
Aspect ratio	0.86 ± 0.08
Drug content (*n* = 3)	93.79 ± 2.55%

Values are the mean ± standard deviation.

**Table 4:** The stability study of the prepared CUBs-MN_D_ under all storage conditions.

Storage Condition	Initial Storage	25 ± 2 °C (60 ± 5% RH)	40 ± 2 °C (75 ± 5% RH)	5 ± 2 °C
Time Point	0 Month	6 Month	6 Month	6 Month
Physical appearance	Microneedles	No Change	No Change	No Change
Drug content (%)	93.79 ± 2.55	92.20 ± 1.42	91.10 ± 1.90	93.02 ± 1.57
Drug Permeation (Jss) μg/cm^2^/h	101.17 ± 2.32	100.65 ± 2.96	99.04 ± 2.17	100.3 ± 2.58

## Data Availability

Data is contained within the article.
